# Six Sigma: Process of Understanding the Control and Capability of Ranitidine Hydrochloride Tablet

**DOI:** 10.4103/0975-1483.76415

**Published:** 2011

**Authors:** AR Chabukswar, SC Jagdale, BS Kuchekar, VD Joshi, GR Deshmukh, HS Kothawade, AB Kuckekar, PD Lokhande

**Affiliations:** *MAEER’s Maharashtra Institute of Pharmacy, MIT Campus, Paud Road, Kothrud, Pune – 411 038, India*; 1 *Department of Production, Glaxo Smith Kline Pharmaceuticals Ltd., Ambad, Nashik – 422 010, India*; 2 *Department of Chemistry, University of Pune, Pune, Maharashtra, India*

**Keywords:** DMAIC, process capability, ranitidine, six sigma

## Abstract

The process of understanding the control and capability (PUCC) is an iterative closed loop process for continuous improvement. It covers the DMAIC toolkit in its three phases. PUCC is an iterative approach that rotates between the three pillars of the process of understanding, process control, and process capability, with each iteration resulting in a more capable and robust process. It is rightly said that being at the top is a marathon and not a sprint. The objective of the six sigma study of Ranitidine hydrochloride tablets is to achieve perfection in tablet manufacturing by reviewing the present robust manufacturing process, to find out ways to improve and modify the process, which will yield tablets that are defect-free and will give more customer satisfaction. The application of six sigma led to an improved process capability, due to the improved sigma level of the process from 1.5 to 4, a higher yield, due to reduced variation and reduction of thick tablets, reduction in packing line stoppages, reduction in re-work by 50%, a more standardized process, with smooth flow and change in coating suspension reconstitution level (8%w/w), a huge cost reduction of approximately Rs.90 to 95 lakhs per annum, an improved overall efficiency by 30% approximately, and improved overall quality of the product.

## INTRODUCTION

Six sigma is a system of practices originally developed to systematically improve processes, by eliminating the defects. The defects are defined as units that are not members of the intended population. Since it was originally developed, six sigma has become an element of many total quality management (TQM) initiatives. Six sigma is a registered service mark and trademark of Motorola, Inc. Motorola has reported over US $17 billion in savings from six sigma, as of 2006. Other companies using this technique are Honeywell International (previously known as Allied Signal) and Raytheon and General Electric (introduced by Jack Welch). In recent times six sigma has been integrated with the TRIZ methodology for problem solving and product design.[1–4]

A process that is six sigma (six sigma process quality is considered as world class quality) will yield just two instances of non-conformances out of every billion opportunities, provided there is no shift in the process average, and the same process will yield 3.4 instances of non-conformances out of every million opportunities with an expected shift of 1.5 sigma in the process average. A process at four sigma levels (considered average process) is expected to yield 63 instances of non-conformances for every million opportunities, without a shift in process average and 6210 instances of non-conformances with a shift in the process average. Contrary to the above, a process at the two sigma level is considered a poor quality process and is expected to yield 3,08,537 instances of non-conformances with the shift of 1.5 sigma in the process.[5–7] The data for the process at different sigma levels are given in Table 1.

**Table 1:** Sigma Table

Sigma	Defects per million	Yield
6	3.4	99.9997%
5	233.0	99.977
4	6.210.0	99.379
3	66.807.0	93.32
2.5	158.655.0	84.1
2	308.538.0	69.150.0
1.5	500.000.0	50.0
1.4	539.828.0	46.0
1.3	579.260	42.1
1.2	617.911.0	38.2
1.1	655.422.0	34.5
1.0	691.462.0	30.9
0.5	841.345.0	15.9
0.0	933.133.0	6.7

Defect values in the Table 1 suggest that as the sigma level goes up the defect rate reduces, which means the product quality improves. Six sigma, therefore, is a powerful tool that can transform defect prone business / industry into an organization of perfection. Thus a journey toward sigma level means a journey toward making fewer and fewer mistakes in everything.

**Figure 1:**
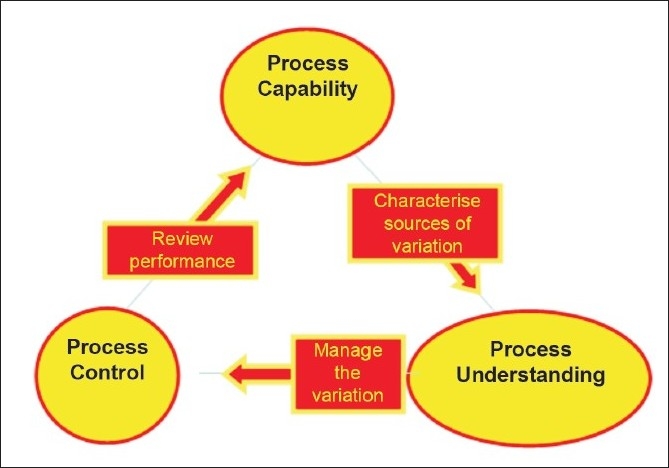
Process capability, control, and understanding framework

**Figure 2:**
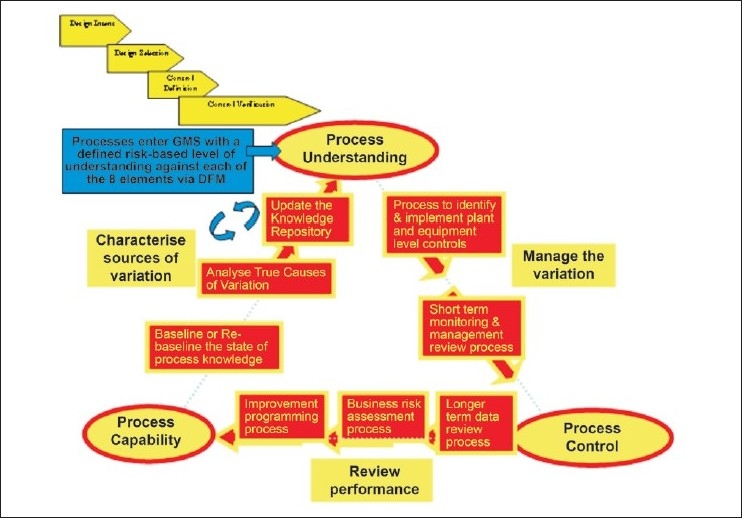
PUCC, eight elements

The PUCC framework is explained in Figure 1. The framework can be used to manage: current processes, process change, and new processes. Eight elements of PUCC[8,9] are shown in Figure 2.

## DMAIC

The basic methodology consists of the following five steps:

- *Define* the process improvement goals that are consistent with customer demands and enterprise strategy.
- *Measure* the current process and collect relevant data for future comparison.
- *Analyze*, to verify the relationship and causality of factors. Determine what the relationship is, and attempt to ensure that all factors have been considered.
- *Improve* or optimize the process based on the analysis, using techniques such as the design of the experiments.
- *Control*, to ensure that any variances are corrected before they result in defects. Set up pilot runs to establish process capability, transition to production, and thereafter continuously measure the process and its capability.[10–12]

## EXPERIMENTAL

### Focus methodology

PUCC stands for three phases, process of understanding, process control, and process capability. These three phases cover the DMAIC methodology of six sigma. Instead of carrying the project in the phases of PUCC, the project was covered by the DMAIC method.[13]

Ranitidine hydrochloride production falls in the following stages: Weighing and blending, Compression, Coating, and Packing.

Forty batches from NL461 – NL500 were monitored throughout these four stages, and enormous data was collected, to cover the measure phase of DMAIC.

The data collected was then analyzed using STATISTICA, MINITAB 14 (STATISTICAL PACKAGES), and MICROSOFT EXCEL.

On completion of the analysis phase, the improved phase is initiated, and then the action plan for the control phase of DMAIC is designed.

### Define

The process improvement goals are consistent with customer demands and the enterprise strategy. The complete process of manufacturing is defined in terms of its various process flow diagrams; the definition of the problem must be stated in this step. Process capability parameters are defined and are critical to the customer and to the quality parameters that are defined.

*Ranitidine hydrochloride* (RHCL) tablet manufacturing is monitored for a long run, up to 35 batches, with data regarding the characterization of raw materials, comparability study of alternative sources of raw materials, manufacturing process such as blending, compression, and packing, packing material characterization, packing line efficiency, and packing line yields. Data has to be collected and treated statistically, to study the trend analysis and define most of the contributing variables in the process variations. The present Sigma level of the overall manufacturing process is between 1.5 and 2.5, and the target Sigma value is 4.

### Baseline of manufacturing process is defined using the following tools

**Figure 3:**
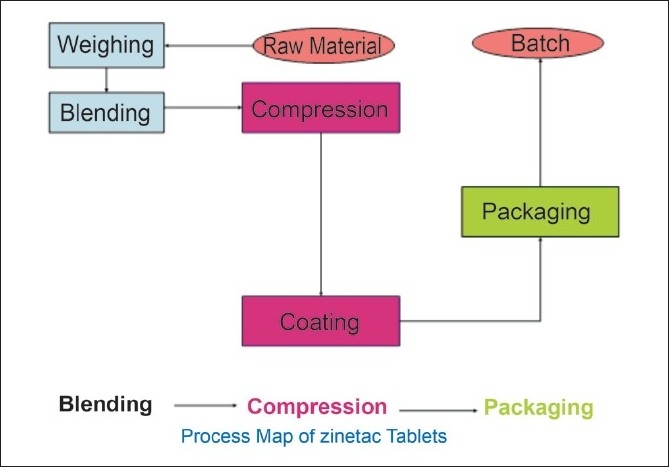
Process map of the RHCL tablet

*The Ranitidine hydrochloride* Process Capability Parameters are, Proposed CTQ Trait, Process Map of RHCL tablets, [Figure 3], Flow diagram for parameters affecting the process, Process flow diagram for RHCL tablets, input process output (IPO) diagram for blending process, IPO diagram for compression process, blending parameters, data required, correlation analysis, variable factor analysis, multiple variable graphs, Pareto charts for variables, line plots, and trend plots.

### RHCL process capability parameters

Critical to Customer (CTC): Defects that would make the customer question the quality or effectiveness of the product.[14]

### Proposed CTC trait

Color (uniformity, right color) legibility of print / embossing, broken / chipped tablets, thick or thin tablets, efficacy, shape.[15]

- Critical to Quality (CTQ): Defects that would cause a batch rejection, batch re-work or FDA action.
- Critical to Process (CTP); an item that if not held within a certain range as determined through process development would cause out-of-specification results.[16]

Batch reconciliation in dispensing (per raw material), blending time parameters met, blending yield, tablet weights (individual, average), tablet thickness, tablet hardness (individual, average), tablet friability, tablet disintegration time, tablet shape, tablet size, debossing, foreign product / material, uncoated tablet, broken tablet, compression accountability, compression yield, QC assay, equilibrium relative humidity, press speed, pre-compression force, main compression force, blending yield, blending accountability, blending LOD, and tablet assay.

### Measure

#### Evaluation of granule[17]

Tables 2 and 3 indicate data for raw material and blending.

**Table 2:** Data for raw materials

B.No.	rr. no.	RHCL net wt	Assay Value of RHCL	Bulk density	Tap Den sity	Carr index	hour	rr no.	MCCP NET weight	Bulk Den sity	Tap density	Carr index	Hausner ratio
nl461	6272	168.9	148.1	0.67	0.74	9.5	1.10	6114	129.7	0.29	0.38	23.7	1.31
nl462	6272	168.9	152.1	0.67	0.74	9.5	1.10	6114	129.8	0.29	0.38	23.7	1.31
nl 465	6272	168.8	149.7	0.67	0.74	9.5	1.10	6114	129.8	0.29	0.38	23.7	1.31
nl 466	6272	168.8	146.5	0.67	0.74	9.5	1.10	6114	129.5	0.29	0.38	23.7	1.31
nl 467	6273	168.8	149.4	0.67	0.74	9.5	1.10	6114	130.05	0.29	0.38	23.7	1.31
nl468	6273	168.8	149.9	0.67	0.74	9.5	1.10	6114	130.4	0.29	0.38	23.7	1.31
nl 469	6273	168.8	146.4	0.67	0.74	9.5	1.10	6114	130.9	0.29	0.38	23.7	1.31
nl 470	6273	168.6	147	0.67	0.74	9.5	1.10	6114	130.9	0.29	0.38	23.7	1.31
nl 471	6273	168.9	150.7	0.67	0.74	9.5	1.10	6114	130.33	0.29	0.38	23.7	1.31
nl 472	6273	168.7	147.8	0.67	0.74	9.5	1.10	6114	130.7	0.3	0.38	21.1	1.27
nl 473	6273	169.4	150.1	0.67	0.74	9.5	1.10	6114	130.4	0.3	0.38	21.1	1.27
nl 474	6274	169.4	151.9	0.67	0.74	9.5	1.10	6114	130.3	0.3	0.38	21.1	1.27
nl475	6274	169.4	151.6	0.67	0.74	9.5	1.10	6115	130.63	0.3	0.38	21.1	1.27
nl476	6274	169.5	146.3	0.67	0.74	9.5	1.10	6115	130.4	0.3	0.38	21.1	1.27
nl477	6274	169.4	151.7	0.67	0.74	9.5	1.10	6115	130.1	0.3	0.38	21.1	1.27
nl 478	6274	169.6	153.6	0.67	0.74	9.5	1.10	6115	130.2	0.3	0.38	21.1	1.27
nl 480	6275	168.4	150.9	0.66	0.73	9.6	1.11	6115	130.4	0.3	0.38	21.1	1.27
nl 481	6275	168.4	150.9	0.66	0.73	9.6	1.11	6115	130	0.3	0.38	21.1	1.27
nl 482	6275	168.7	151	0.66	0.73	9.6	1.11	6115	130.2	0.3	0.38	21.1	1.27
nl 483	6275	168.7	150.6	0.66	0.73	9.6	1.11	6115	130.3	0.3	0.38	21.1	1.27
nl 484	6274	168.6	146.2	0.67	0.74	9.5	1.10	6757	130.1	0.3	0.4	25.0	1.33
nl 485	6474	168.9	150.9	0.67	0.74	9.5	1.10	6757	130.1	0.3	0.4	25.0	1.33
nl 486	6474	168.9	145.8	0.67	0.74	9.5	1.10	6757	129.9	0.3	0.4	25.0	1.33
nl 487	6474	168.9	150.5	0.67	0.74	9.5	1.10	6757	130.1	0.3	0.4	25.0	1.33
nl 488	6474	169	150.3	0.67	0.74	9.5	1.10	6757	130.5	0.3	0.4	25.0	1.33
nl489	6474	168.9	149.2	0.67	0.74	9.5	1.10	6757	130.8	0.3	0.4	25.0	1.33
nl 490	6475	168.9	149.8	0.67	0.71	5.6	1.06	6757	130	0.3	0.4	25.0	1.33
nl 491	6475	168.6	149.9	0.67	0.71	5.6	1.06	6757	129.9	0.3	0.4	25.0	1.33
nl 492	6475	168.9	154.1	0.67	0.71	5.6	1.06	6757	130.7	0.3	0.4	25.0	1.33
nl 493	6475	169.3	153.4	0.67	0.71	5.6	1.06	6757	130.1	0.3	0.4	25.0	1.33
nl 493	6475	169.2	149.3	0.67	0.71	5.6	1.06	6757	129.7	0.3	0.4	25.0	1.33
nl 495	6475	169	147.7	0.67	0.71	5.6	1.06	6757	130	0.3	0.4	25.0	1.33
n 1 496	6475	169	148.5	0.67	0.71	5.6	1.06	6757	130	0.3	0.4	25.0	1.33
nl 497	6475	169.1	153.4	0.67	0.71	5.6	1.06	6757	130.2	0.3	0.4	25.0	1.33
nl 498	6476	169.9	153.3						130.2	0.3	0.4	25.0	1.33
nl 499	6476	169.2	151.2						130	0.3	0.4	25.0	1.33
nl 500	6476	169.2	153.8						130.4	0.3	0.4	25.0	1.33

**Table 3:** Data on Blending

B no.	Speed of blender in rpm	Time for rotation in minutes	Weight after blending in kgs	Total time for blending in minutes	Weight loss in blending in kgs
NL 461	24	15	299.45	17:12:15	1.4
NL 462	24	15	326.15	16:19:43	1.1
NL 465	24	15	316.8	16:12:21	1.05
NL 466	24	15	299.5	18:40:07	1.025
NL 467	24	15	301.6	16:34:34	-0.5
NL 468	24	15	315.75	16:45:54	2.3
NL 469	24	15	301.2	17:52:45	0.75
NL 470	24	15	300.5	16:07:39	1.25
NL 471	24	15	300.4	19:56:32	1.12
NL 472	24	15	302.75	17:23:34	-1.1
NL 473	24	15	319.5	17:56:03	1.9
NL 474	24	15	327.95	16:49:32	0.7
NL 475	24	15	318.7	16:54:54	1.58
NL 476	24	15	299.8	18:10:02	2.35
NL 477	24	15	299.45	17:10:34	2.3
NL 478	24	15	300.3	16:54:31	1.75
NL480	24	15	318.95	18:59.0	0.6
NL481	24	15	323.7	18:12:21	0.85
NL482	24	15	299.34	16:39:27	1.81
NL483	24	15	299.75	16:50:10	1.5
NL484	24	15	328.85	17:52:10	0.1
NL485	24	15	317.5	17:55:02	2.75
NL486	24	15	299.6	18:16:10	1.45
NL487	24	15	299.9	17:56:03	1.35
NL488	24	15	300.2	16:46:38	1.55
NL489	24	15	299.65	18:13:10	2.3
NL490	24	15	299.95	17:30:30	1.2
NL491	24	15	299.91	17:40:40	0.84
NL492	24	15	300.75	16:30:40	1.1
NL493	24	15	300.05	17:40:50	1.6
NL494	24	15	301.15	18:10:19	1.1
NL495	24	15	322.9	18:07:20	1.85
NK496	24	15	323.75	17:55:33	2.4
NL497	24	15	322.5	17:54:53	1.05
NL498	24	15	326.55	18:05:10	0.8
NL499	24	15	330.15	16:56:10	0.3
NL500	24	15	318.85	17:55:10	1.2

#### Evaluation of tablet[18]

The features evaluated were: Tablet thickness and diameter, tablet hardness, friability, uniformity of weight, and uniformity of content.

#### Analyze

Data collected from 38 batches was analyzed on a statistical tool called ‘Statistica’.

Data was analyzed for the following phases and in the following order:

- Raw material and blending
- Compression
- Coating
- Packing

Verification of relationships and causality of factors were carried out by using various statistical tools.[19–21] What the relationship was also determined and an attempt was made to ensure that all factors had been considered. (All representative figures of each phase mentioned above had been attached in the same sequence).

#### Improve

The process was improved or optimized based on the analysis, using techniques like design of experiment[22] and so on. With the help of the above analysis done by various statistical tools and techniques, various UDEs (undesirable effects) were discovered, and the severity and causes of these UDES were discussed. Desirability of various improvements was checked and certain suggestions were made for improvements, which were then discussed. Subsequently, these improvements would be implemented and their impact would be observed on the improvement of yield and sigma level. The major U.D.E’s discovered during the analysis phase and their suggestion for improvement made the process more capable and robust.

#### Control

All actions taken in the above-mentioned four phases should remain in control, that is, they should be sustained. An action review is important for that, and this was carried out in this phase.

## RESULT AND DISCUSSION

Undesirable effects were observed during the analysis of the process using different statistical tools. For minimization of undesirable effects, various changes in terms of process alteration and corrective measures for manual handling of the process were made in the process, for making it more capable and robust.

In order to improve process capability, in the following stages different parameters are targeted and their exact role is discussed.

- Blending
- Compression
- Coating
- Packing

(All representative figures of each phase mentioned above have been attached in the same sequence).

### Blending

**Figure 4:**
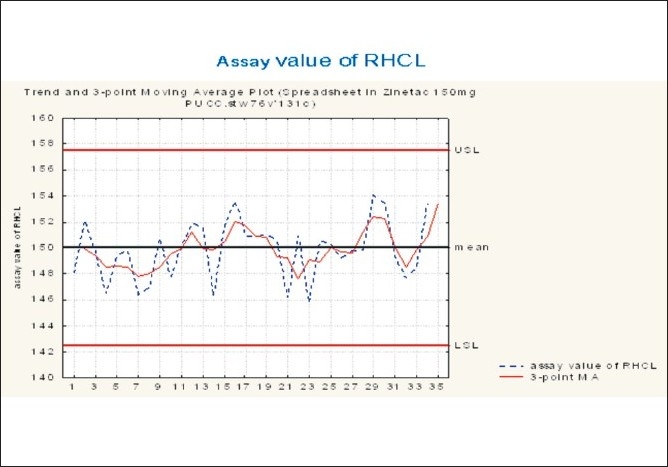
Trend plot for assay value of RHCL in the blending phase

**Figure 5:**
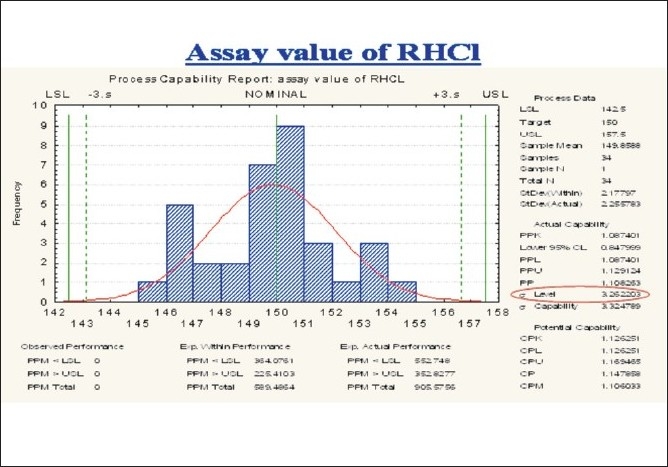
Process capability report of the assay value of RHCL in the blending phase

During the blending process, assay variation [Figures 4 and 5] was observed in the range of 146 – 152. In order to overcome this variation, the existing blender had to be replaced with a new blender of higher capacity, it was validated and a measurement system analysis of the blend was performed.

Particle size distribution of Ranitidine Hydrochloride (RHCL) and microcrystalline cellulose (MCCP) was not available to manufacturing heads. This had been informed to the Manufacturing Department on the browser, for RHCL as well as MCCP, from the Quality Assurance Department, with the help of the Information Technology Department.

**Figure 6:**
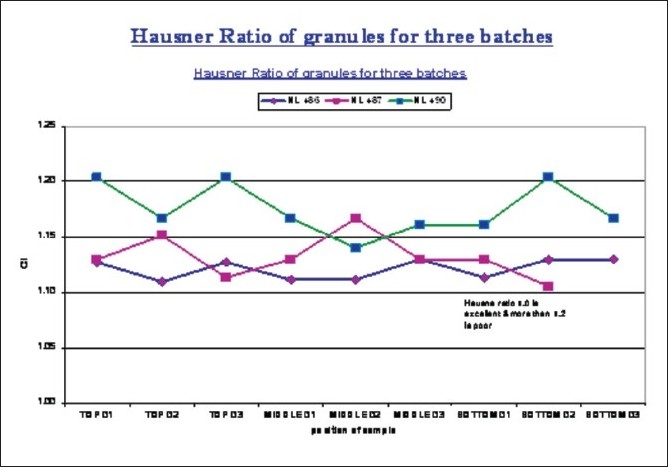
Hausner ratio of granules for three batches in the blending phase

**Figure 7:**
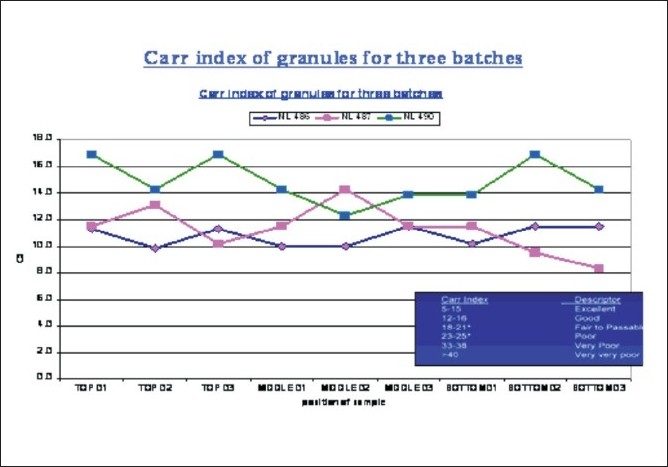
Carr index of granules for three batches in the blending phase

High cycle time for the activity of weighing, sifting, and blending was required and more manpower was used in this stage for weighing, sifting, and blending. To avoid this, load charting of the weighing, sifting, and blending stage had to be carried out, for minimization of manpower, Recalculation of the cycle time at the installation of the new blender was carried out. Parameters for the granule evaluation are as shown in Table 4 and Figures 6 and 7.

**Table 4:** Data on Granules

Sr. no	NL 486					NL 487					NL 490				
	Bulk density	Tapped density	Hausner ratio	CI	Description	Bulk density	Tapped density	Hausner ratio	CI	Description	Bulk density	Tapped density	Hausner ratio	CI	Description
Top 01	0.55	0.62	1.13	11.3	E	0.54	0.61	1.13	11.5	E	0.54	0.65	1.20	16.9	F
Top 02	0.55	0.61	.11	9.8	E	0.53	0.61	1.15	13,1	G	0.54	0.63	1.17	14.3	G
Top 03	0.55	0.62	1.13	11.3	E	0.53	0.59	1.11	10.2	E	0.54	0.65	1.20	16.9	F
Middle 01	0.54	0.6	1.11	10.0	E	0.54	0.61	1.13	11.5	E	0.54	0.64	1.17	14.3	E
Middle 02	0.54	0.6	1.11	10.0	E	0.54	0.63	1.17	14.3	G	0.57	0.65	1.14	12.3	E
Middle 03	0.54	0.61	1.13	11.5	E	0.54	0.61	1.13	11.5	E	0.56	0.65	1.16	1.16	E
Bottom 01	0.53	0.59	1.11	10.2	E	0.54	0.61	1.13	11.5	E	0.56	0.65	1.16	13.8	E
Bottom 02	0.54	0.61	1.13	11.5	E	0.57	0.630	1.11	9.5	E	0.54	0.65	1.20	16.9	F
Bottom 03	0.54	0.61	1.13	11.5	E	0.55	0.61	1.09	8.3	E	0.54	0.63	1.17	14.3	G

**Table:** 

Carr Index	Descriptor
5 – 15	Excellent
12 – 16	Good
18 – 21	Fair to passable
23 – 25	Poor
33 – 38	Very poor
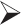 40	Very very poor

**Figure 8:**
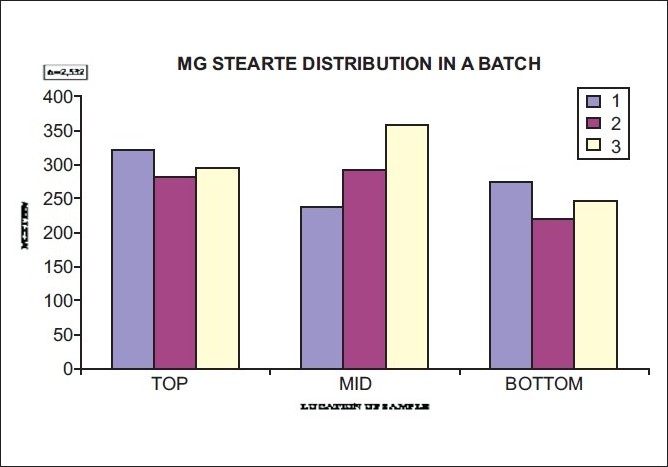
Distribution of magnesium stearate in a batch, in the blending phase

Magnesium stearate [Figure 8] distribution in the batch at the top, middle, and bottom was also observed. For even distribution of magnesium stearate, the angle of repose studies was carried out at the time of installation of the new blender.

### Compression

**Figure 9:**
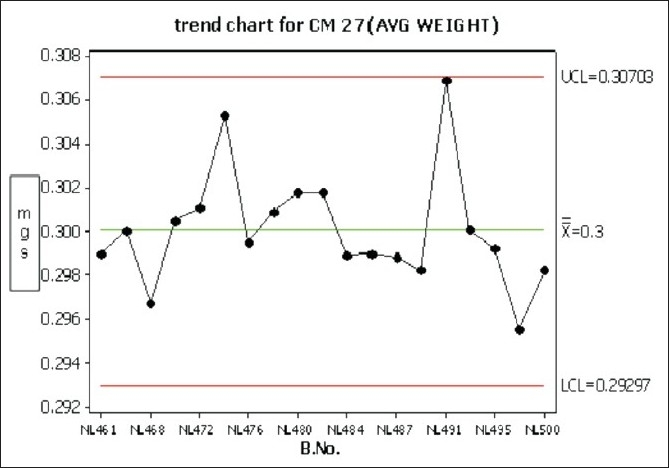
Trend chart for average weight in the compression phase

**Figure 10:**
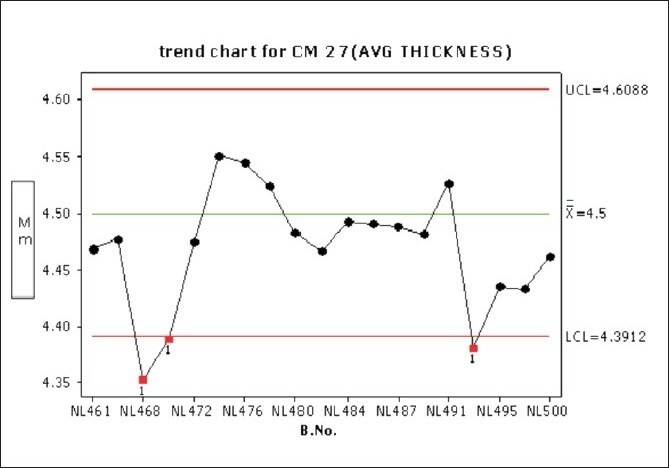
Trend chart for average thickness in the compression phase

**Figure 11:**
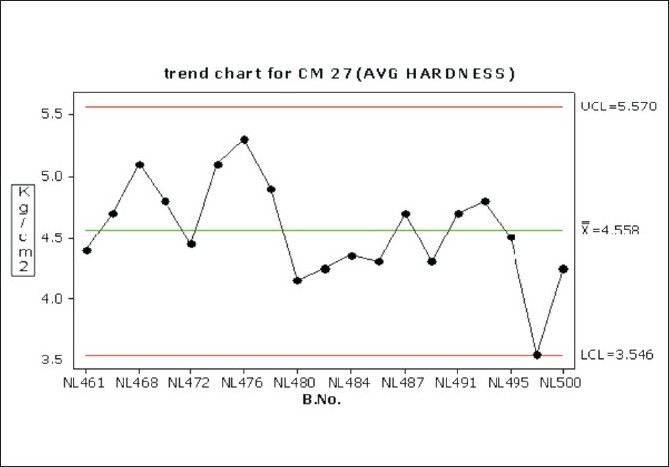
Trend chart for average hardness in the compression phase

**Figure 12:**
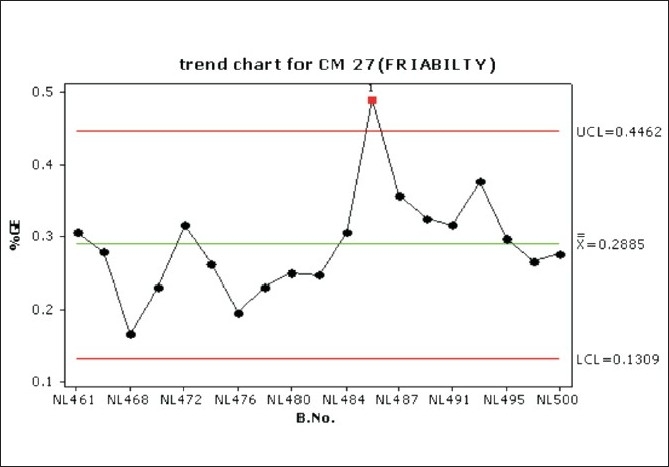
Trend chart for friability in the compression phase

**Figure 13:**
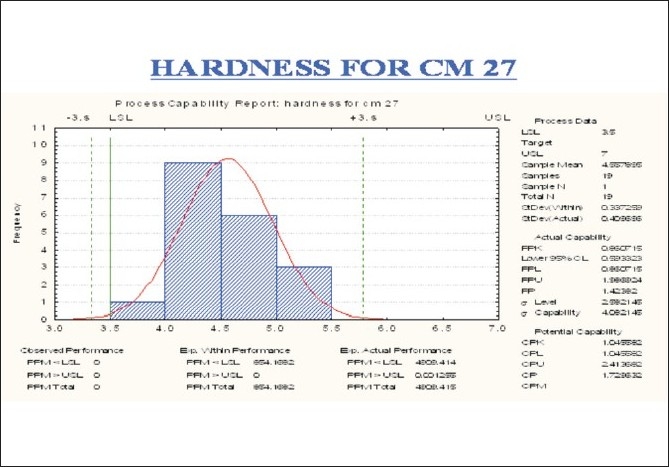
Process capability report of hardness in the compression phase

Considerable variation in tablet weight, tablet thickness, and tablet hardness was observed in Table 5, Figures 9 –13.

**Table 5:** Compression Data

Compression on CM 26						
B. no.	m c no	Avg. weight in gms	Avg. thickness in mms	Avg hardness Kg/cm^2^	Friability in %	UR generated In kgs
NL 462	cm 26	0.2997	4.52673	4.9	0.203	8.9
NL 465	cm 26	0.3017	4.47627	4.2	0.231	11.23
NL467	cm 26	0.2979	4.46443	4.75	0.197	8.98
NL469	cm 26	0.3023	4.4972	4.95	0.222	12.2
NL471	cm 26	0.3011	4.5111	5.15	0.108	8.67
NL473	cm 26	0.3026	4.48853	4.55	0.197	9.08
NL475	cm 26	0.3025	4.438	4.95	0.228	13.9
NL477	cm 26	0.2995	4.40651	5.2	0.26	8.02
NL481	cm 26	0.3003	4.46448	4.55	0.358	9
NL483	cm 26	0.2998	4.45533	4.25	0.311	9
NL485	cm 26	0.3013	4.4815	4.4	0.299	8.552
NL488	cm 26	0.2968	4.4744	4.3	0.268	9.8
NL490	cm 26	0.2957	4.459	4.55	0.299	9.23
NL492	cm 26	0.2997	4.486	4.65	0.292	11.72
NL494	cm 26	0.3021	4.3963	5.2	0.323	9.23
NL496	cm 26	0.2914	4.436	3.15	0.221	10.8
NL498	cm 26	0.299	4.464	4.45	0.244	9.4
NL499	cm 26	0.3005	4.4455	4.3	0.26	12.8

In order to solve the variation in the compression stage of different machines, data was observed and entered simultaneously on a run chart after replacement of a punch set. Checking the thickness of the tablets on the first two rotations of the machine, every time the machine was restarted, and also collection of data on punch height was done.

Average U.R (Utilizable Residue) produced per batch was 3.5%, that led to extra man hours for rework. In order to overcome this variation, interlinking the speed of the machine and force feeder was done and also inspection of whether the tablets were taken out, each time the machine was adjusted or not was checked.

Considerable variation in friability was found and in order to solve this variation, monitoring of moisture content was done regularly and data was generated for CHEMFILED, to compare it with the existing RANQ.

More unaccounted time and minor stoppages were observed and to minimize this, Time value mapping of the cleaning activity on a daily and weekly basis was done and operator attitude and awareness was addressed.

Rotation of the machine operator was observed daily. To minimize this, the staff was fixed for a period of two weeks.

Time wasted while the first shift ended (30 minutes closing time) and the second shift started (15 minutes starting time) was observed. To utilize that time, overlapping between these two times were challenged.

The Present Overall Equipment Effectiveness (OEE) is 28.75%, and 28.30% for Compression machine CM26 and CM27 was observed. To improve the Overall Equipment Effectiveness (OEE) level up to 45% for CM26 and CM27 as a first target, the project had to be taken by the manufacturing heads.

### Coating

**Figure 14:**
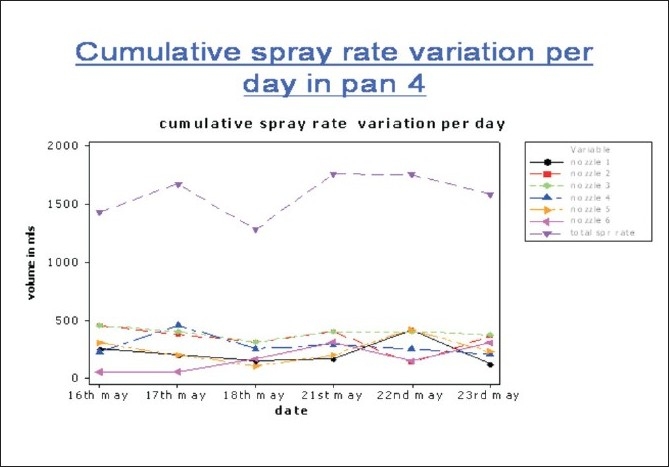
Cumulative spray rate variation per day in pan 4 of the coating phase

**Figure 15:**
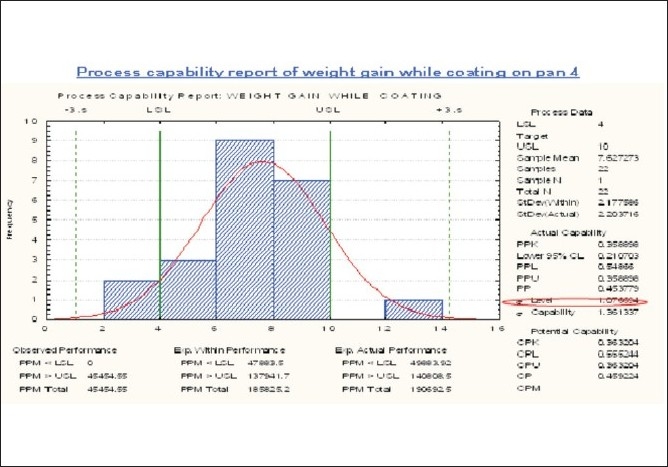
Process capability report of weight gain while coating on pan 4 in the coating phase

Considerable cumulative Spray rate variation [Tables 6 and 7], Figures 14 and 15 as well as individual gun spray rate variation (50 ml – 450 ml) was observed, in order to solve this variation, Gun maintenance and replacement was done and when required chocking of guns to be minimized or eradicated and also gun cleaning frequency and its effectiveness to be addressed.

**Table 6:** Data on coating spray rate

Date	Coating pan	Nozzle 1	Nozzle 2	Nozzle 3	Nozzle 4	Nozzle 5	Nozzle 6	Total spray	Avg. spray	Spray time
16 May	4	250	450	450	230	300	50	1430	238.3333	115.3846154
17 May	4	200	375	400	450	200	50	1675	279.1667	98.50746269
18 May	4	150	310	310	250	100	160	1280	213.3333	128.90625
21 May	4	160	400	400	290	200	310	1760	293.3333	93.75
22 May	4	410	130	400	250	410	150	1750	291.6667	94.28571429
23 May	4	120	360	370	210	225	300	1585	264.1667	104.1009464

**Table 7:** Coating data on pan no. 5

B. no.	Coating pan no.	Total coating time	Drying time	Tablet bed temp.	Cfm of inlet air	Cfm of outlet air	Inlet air temp tange set	Inlet air temp. min actual	Inlet air temp. max actual	Outlet air temp. min	Outlet air temp. mix	Spray rate	Erh after drying	Weight gain while coating
NL466	5	203	53	60.4	4800	8400	62	60	63	50	52	1440	4.6	7
NL469	5	230	72	59	4250	8175	62	63	65	50	53	1430	4.5	3.5
NL471	5	200	50	54	4260	8175	72	73	74	50	53	1430	5.8	6.5
NL475	5	190	40	57	4800	8400	62	58	62	48	52	1710	5.6	5.6
NL477	5	190	42	59	4800	8175	68	72	73	48	50	1710	6.5	6.5
NL481	5	220	60	50	4250	8175	80	69	71	42	45	1434	4.4	6.2
NL483	5	190	42	54.5	4250	8400	75	73	75	45	46	1434	3.5	7
NL486	5	220	53	59	4800	8400	62	61	63	50	53	1620	6.8	4
NL488	5	200	54	58.9	4800	8400	62	60	62	50	53	1620	4.6	8
NL492	5	215	65	59.5	4250	8175	62	62	63	50	53	1220	1.4	17.4
NL494	5	175	35	61	4250	8175	62	63	64	50	53	1220	4.4	4
NL496	5	220	63	60	4250	8175	62	60	62	51	54	1220	5.3	6
NL498	5	230	78	61	4260	8175	62	61	63	49	40	1300	2.3	6
NL500	5	210	55	60	4260	8175	62	60	64	49	52	1300	7.2	8

Considerable variation in the parameters like: inlet air temperature, outlet air temperature, inlet air cfm, outlet air cfm, tablet bed temperature, and so on were observed. In order to minimize these variations, the same parameters were kept in PLC for both the coating pan and calibration of velocity. The sensor for filter cleaning was done. Also standard parameters values were set.

No robust method of measurement was available to measure gun distance from tablet bed, In order to solve this problem; Collection of data to see the validated results was done.

Uneven weight gain while coating was observed and to solve this variation in weight gain, interaction of controllable parameter in coating that results in more or less weight gain, for e.g. spray rate, inlet and outlet air temp, inlet and outlet air cfm, atomizing air pressure was observed.

Present Overall Equipment Effectiveness (OEE) is 34.48% and 23.66% for coating pan4 and coating pan5, respectively. In order to improve the Overall Equipment Effectiveness (OEE) level up to 50 - 55% for both the pans as a first target, the project was taken up by manufacturing heads.

There was need felt to apply Design of experiment (DOE) for change in coating suspension Reconstitution Level. In order to carry out the six sigma tool, design of experiment (DOE) for change in coating suspension Reconstitution Level, a separate project had to be handled. For that an STP (Situation, Target, and Plan) was prepared and also a trial protocol prepared.

### Packing

**Figure 16:**
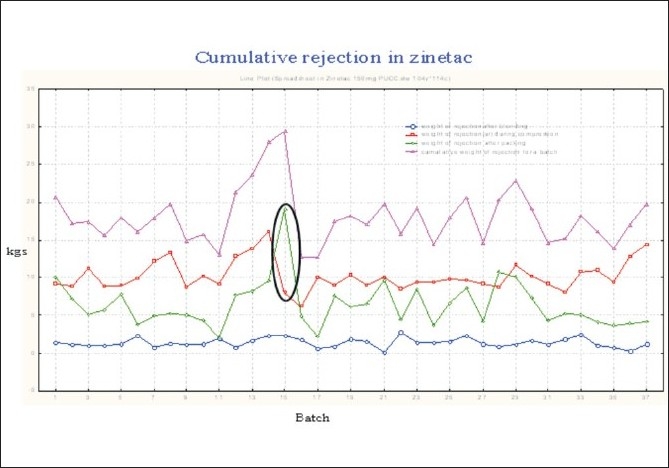
Cumulative rejection of RHCL in the packing phase

The Average Overall Equipment Effectiveness (OEE) of packing lines [Table 8, Figure 16] was 40.53% (single shift bases), with individual line OEE being: line 1 (39.39217%), line 2 (46.18117%), line 3 (39.16551%), line 4 (35.52693), and line 6 (42.38882%).

**Table 8:** Packing data on line no. 1

B. no.	Line No.	Total working time for batch	Temp of left roller min	Temp of left roller min	Temp of left roller min	Temp of left roller min	Weight transferred from coating	Stoppages (in mins)						Weight of tablet after defoiling	Weight of defoiled strip and blank strip	Run time in mins	Unacco-unted time
								For M/c adjustment	Roll change	Printing or othr problem	Tablet problem	Breaks	Total stoppage in mins				
NL 461	1	691	133	179	148	170	316.5	34	36	30	61	77	233	1003	15.57	453	73
NL 462	1	575	143	171	139	182	294.6	10	41	5	30	90	176	563	10.148	400	20
																	
NL 473	1	535	143	167	139	220	310.4	15	30	2	21	75	148	21	3.85	395	15
NL 477	1	843	160	182	154	189	307.9	36	39	20	74	130	299	1903	21.8	544	164
NL 482	1	542	151	186	157	177	293.1	14	31	10	15	75	145	609	775	397	17
NL 489	1	594	145	182	144	179	295.1	4	36	19	11	80	150	86	13.44	444	64
NL 493	1	544	145	174	147	160	297.7	23	27	9	9	75	143	735	9.44	401	21
NL 500	1	545	135	165	148	170	315.15	7	36	4	8	70	123	415	7.97	422	42

In order to achieve Overall Equipment Effectiveness (OEE) level in the range of 50 - 65% for all the packing lines as a first target, the project had to be taken by the Packing Department heads.

It has been observed that the Roll change time contributes to 25 – 34% of total stoppage time while completing a batch. To minimize this time period, the procedure was standardized for changing the rolls. Also efforts were made by the Engineering. Department to bring automation in the process of roll change and the process mapping was carried out at least once in a fortnight.

A run time (on a single shift basis of 570 minutes) of 320 minutes was observed and to increase this run time, the stoppages due to machine adjustment, tablet problem, and other miscellaneous factors were minimized. Also process mapping was carried out once in a month. Mapping of micro-stoppages, morning–evening tea breaks, and ground level exit was carried out, to minimize unaccounted time, and a BRAVO CARD system was implemented, to give recognition to the line operator who was providing the maximum output in a week.

Loose winding of plane and printed rolls were observed. In order to solve this problem, the issue was addressed at the time of procurement of the rolls from the supplier.

The average foil rejection per batch was 12 kg and the average weight of the defoiled tablets was 6 kg per batch. [Figure 16]. To minimize this rejection, the variation in tablet thickness, hardness, weight, and coating, was reduced, which made the compression and coating stage more robust, to produce minimum number of defects. The operator attitude and awareness was also addressed, and machine issues that led to tablet and foil rejection, were taken care of by the Engineering Department.

## CONCLUSION

As various UDEs (Undesirable effects) were discovered and discussed in the analysis, an improved phase of DMAIC, recommendations, and suggestions came about, to make the present process more robust against defects, either by bringing new steps in the process or by improving the same existent current process. This will result in benefits, some tangible and some non-tangible.

- Given here are some value additions from the process of RHCL production obtained by the implementing of PUCCInstallation of high capacity blender of 1000 kg, replacing the current 300 kg blender, thus saving the man-hours by 66% (approximately).
- Introducing high capacity tote-bins of 200 – 300 kgs, which would result in reducing the unloading time from the blender, coating the pan to one-third. Man and material motion would be reduced to one-third. Time of loading and unloading of tote-bins, to lifts, and to and from the mezzanine floor would be one-third. Batch changeover time would be reduced to 33% of the current time, and hence, less amount of U.R would be generated.
- OEE improvement for compression, coating, and packing stage, which would lead to 30 – 35% increase in OEE for RHCL 150 MG tablets production.
- Set up time reduction for the whole process by 40 – 50%.
- Release of 20 – 30% of the manpower.
- Process waste reduction, both in compression and packing by 35%
- Rework reduction by 50 – 70%.
- Reduction in packing line stoppages.
- Improved process capability due to improved sigma level.
- A more standardize process.
